# Does Joint Care Impact Teenage and Young Adult’s Patient-Reported Outcomes After a Cancer Diagnosis? Results from BRIGHTLIGHT_2021

**DOI:** 10.3390/cancers17233868

**Published:** 2025-12-02

**Authors:** Lorna A. Fern, Elysse Bautista-Gonzalez, Julie A. Barber, Jamie Cargill, Richard G. Feltbower, Laura Haddad, Maria Lawal, Martin G. McCabe, Safia Samih, Louise Soanes, Dan P. Stark, Cecilia Vindrola-Padros, Rachel M. Taylor

**Affiliations:** 1 Cancer Clinical Trials Unit, University College London Hospitals NHS Foundation Trust, London NW1 2PG, UK; 2 Centre for Nurse, Midwife and AHP Led Research (CNMAR), University College London Hospitals NHS Foundation Trust, London NW1 2PG, UK; 3 Department of Statistical Science, University College London, London WC1E 6BT, UK; 4 Managed Service Network for Children and Young People with Cancer in Scotland, Kings Cross Hospital, Dundee DD3 8EA, UK; 5 Leeds Institute for Data Analytics, School of Medicine, University of Leeds, Leeds LS2 9JT, UK; 6 Patient Representative, BRIGHTLIGHT Young Advisory Panel; 7 Division of Cancer Sciences, The University of Manchester, Manchester M13 9PL, UK; 8 Teenage Cancer Trust, London WC1V 7AA, UK; 9 Leeds Institute of Medical Research at St James’s, Leeds LS9 7TF, UK; 10 Rapid Research Evaluation and Appraisal Lab (RREAL), Department of Targeted Intervention, University College London, London WC1E 6BT, UK

**Keywords:** cancer, young people, teenagers, young adults, BRIGHTLIGHT, quality of life, patient-reported outcomes

## Abstract

Background: Healthcare policy in the United Kingdom recognizes that teenagers and young adults (TYA:16–24 years at diagnosis) require specialist care. In England, Principal Treatment Centers (PTCs) exist, delivering enhanced care exclusively within the PTC or as ‘joint care’ with designated hospitals (DHs). We examined whether joint care impacted patient-reported outcomes when compared to care at one site. Methods: A cross-sectional survey was conducted in England, Scotland, and Wales. This included validated measures of quality of life (QoL), anxiety and depression, health status, social support, and illness perception. Comparisons were made based on young people’s exposure to specialist care within 6 months of diagnosis, defined as care in a TYA PTC: all-TYA-care (all care in a TYA unit), no-TYA-care (no care in a TYA unit, care delivered in a children’s/adult unit only), and joint-care (care in a TYA-PTC and in a children’s/adult unit). Results: Overall, 260/1009 (25.8%) participants responded (England n = 241; Scotland/Wales n = 19). Due to different healthcare policies, statistical analysis was applied to England only. Mean QoL scores were <69.7, the threshold indicating impaired QoL (mean 58.65, standard deviation 20.13). After adjustment for confounding factors, no clinically significant differences in mean QoL between categories existed. The adjusted mean difference for all-TYA-care (n = 66) versus no-TYA-care (n = 89) was −2.28 units (95% confidence interval (CI: −8.85 to 4.29) and for joint-care versus no-TYA-care (n = 85), −4.35 units (CI: −10.34 to 1.63). Similarly, no notable differences in social support, anxiety, depression, or illness perception between categories existed. Patients receiving all-TYA-care had a lower average health status compared with no-TYA-care (difference in means −0.09 (CI:−0.18 to −0.01). Conclusions: No evidence that patient-reported outcomes differ by categories of care existed. This may suggest the evolution of TYA cancer services has led to similar outcomes regardless of care category. However, given the small sample size it is not possible to draw firm conclusions.

## Simple Summary

Teenagers and young adults (TYA, aged 16–24) with cancer in the UK are recommended to receive specialist care. In England, this can happen entirely within a Principal Treatment Center (PTC), through a mix of PTC and local hospital care (joint care), or only in children’s or adult units (no TYA care). This study looked at whether the type of care affected patient-reported outcomes such as quality of life, mental health, and social support. A survey was completed by 260 out of 1009 young people, mostly from England. Quality of life scores were generally low, but after adjusting for other factors, there were no significant differences between care types. Similarly, anxiety, depression, and social support did not differ. One small difference in health status was found but may not be meaningful. Overall, care type did not appear to affect outcomes, but the small number of responses means strong conclusions cannot be made.

## 1. Introduction

In the United Kingdom, approximately 2110 new cancer diagnoses occur in teenagers and young adults (TYA) aged 15–24 years annually [1]. Cancer is the most common cause of non-accidental death in TYA, accounting for 9% of deaths in males and 15% of deaths in females [2]. Young people present with a range of cancers, many of which are rare in the general population [3]. Five-year survival for the main cancer types occurring in young people varies from 60 to 93%; however, there is considerable variation and unmet clinical need in some subtypes such as Glioblastoma where no young people are expected to survive beyond 5 years [4]. Historically, survival improvements have lagged behind those observed for adult patients [5]. It was this disparity in survival improvements that underpinned the emergence of TYA cancer care as an international specialty. Furthermore, specialist care for this group is critical as TYA treatment-related morbidity is considerable, as are interruptions to social development, education, and employment [6,7,8,9,10], highlighting the importance in considering non-clinical outcomes, such as quality of life (QoL) and patient-reported outcomes [7].

The impact of cancer during adolescence and young adulthood presents significant problems for young people and the healthcare systems trying to care for them. Neither children nor adult services are suitably equipped to handle the range of cancer types that young people present with nor the unique psychosocial needs that characterize this age group. There is international recognition that TYA merit specific age-appropriate care catered to their unique needs [11,12,13]. Effective care allowing young people to transition back to as healthy a life as possible requires site-specific cancer expertise and care from a wider group of TYA cancer professionals who are experts in caring for young people as well as cancer, including but not limited to, psychologists, social workers, and youth support coordinators.

Different models of delivering care for young people exist internationally [13,14,15,16,17,18,19,20] and in England this is centered around care in 13 dedicated Principal Treatment Centers (PTCs). The model of care therefore includes an age-appropriate environment, a nursing and supportive care workforce to deliver care, integrated psychosocial, and medical care, and processes for entry into clinical trials and fertility preservation. In England these were first defined in the National Institute for Health and Care Excellence (NICE) Improving Outcomes Guidance in 2005 [21]. This mandated a model of ‘age-appropriate care’ where all those diagnosed under 19 years of age were to receive all of their care in a TYA-PTC and those aged 19–24 should be offered ‘unhindered access to age-appropriate care’ and a choice as to whether to have their care delivered in the TYA-PTC or more locally to home. Three groups of patients therefore exist: those who had all their care in the TYA-PTC, those who had no care in the TYA-PTC (either in children’s or adult cancer services), and those who had some care in the TYA-PTC and some care in an adults’ or children’s cancer service. Young people who had access to the TYA-PTC either for all or some of their treatment would therefore have access to a full model of care, as described above, while those who did not, received standard cancer and supportive care only. Similar models of care now exist in Scotland and Wales, where specialist TYA Principal Treatment Centers are linked to other hospitals delivering some or all care [22,23].

The TYA-PTC model, described as ‘age-appropriate care’ in the NICE Improving Outcomes Guidance [21] lacked rigorous prospective evidence that care delivered in this way improved outcomes, which outcomes it may influence, or the associated costs to the patient or the National Health Service (NHS). Subsequently, we conducted a national longitudinal evaluation of the TYA-PTC model of care as described in the NICE Improving Outcomes Guidance, known as the BRIGHTLIGHT study [24]. Central to this mixed methods program of research was a cohort study which aimed to measure how QoL and other clinical outcomes were influenced by the extent of exposure to TYA-PTC care in the three years after diagnosis [25,26,27]. When looking at the three groups of patients, recruited between 2012 and 2014, and after adjustment for key confounders, outcomes including QoL, patient-reported outcomes, and costs favored those who had no-TYA-PTC care most and the some-TYA-PTC group least [24]. The mean QoL score for those being treated in no-TYA-PTC was 5.63 (95% confidence interval (CI) 2.77 to 8.49) points higher when compared with young people receiving some-TYA-PTC care, and 4.17 points higher (95% CI 1.07 to 7.28) compared with all-TYA-PTC care. Differences were greatest 6 months after diagnosis, reduced over time, and did not meet the 8-point level that is proposed to be clinically significant [27]. Rates of improvement of QoL were superior in the all-TYA-PTC and some-TYA-PTC patient groups. Despite extensive testing, we identified no patient or disease factors that explained why the some-TYA-PTC group have consistently inferior outcomes compared with the no-TYA-PTC or all-TYA-PTC groups. However, the cohort represented 20% of the total population of TYA diagnosed during the recruitment period [28], and professional gatekeeping was identified as a considerable barrier to recruitment.

New commissioning guidance prepared by the NHS England Specialized Commissioners in 2019 retained the concept of care delivered in part by TYA-PTCs and in part by other hospitals, now described as ‘joint care’, supported by regional operational delivery networks to coordinate care across NHS services within a region. This was with the aim that young people would have access to a specialist TYA workforce wherever they were treated and more coordinated care in a joint care model [29]. This was proposed prior to the BRIGHTLIGHT results being released, which also demonstrated that the culture of TYA care requires time to develop [30,31]. We surmised that patients identified as experiencing ‘joint care’ may have different outcomes to the some-TYA-PTC group in the 2012 BRIGHTLIGHT cohort. We therefore hypothesized that, over the last decade, the culture of TYA cancer care would have become more embedded in routine practice, with greater coordinated care, and that the difference in outcomes reported in the BRIGHTLIGHT cohort would no longer be evident. The aim of this study was to determine if there were clinically significant differences in patient-reported outcomes for young people with cancer receiving ‘joint care’ in England during 2022 compared to all or no care in a TYA-PTC. We also aimed to include Scotland and Wales and additionally examine whether a direct approach from the research team to patients, not involving their treating healthcare teams, would increase the recruitment rate compared to the original 2012 BRIGHTLIGHT cohort.

## 2. Materials and Methods

### 2.1. Study Design

BRIGHTLIGHT_2021 was a convergent parallel mixed methods study consisting of a cross-sectional survey and a rapid ethnography study. Results from the survey are reported here, which followed the first wave of data collection in the 2012 BRIGHTLIGHT study [27]. HES data were not available in the current study and therefore young people’s self-reported main place of care within the first 6 months of diagnosis was used as an alternative (this was tested with the 2012 BRIGHTLIGHT cohort data, which gave similar results). Comparisons were therefore made between the main place of care being a specialist young person’s ward/unit (all-TYA-PTC), pediatric or adult ward/unit (no-TYA-PTC), or joint care for those treated in a TYA ward/unit who indicated they also received care in another hospital. These comparisons therefore accounted for the place where care was delivered, not the care that was received.

### 2.2. Patient and Public Involvement

BRIGHTLIGHT was developed with young people across the study life cycle of study inception, operation, data interpretation, and dissemination [22]. We enlisted our original BRIGHTLIGHT Young Advisory Panel (YAP) [32] to work with us as co-researchers in BRIGHTLIGHT_2021. Two YAP members joined the research group as co-applicants (LH/ML). Data was collected through an adaptation of the wave 1 BRIGHTLIGHT survey using a bespoke survey that contained five validated questionnaires and 169 descriptive questions related to cancer care experience [33]. This was designed as a Computer-Assisted Personal Interview (CAPI) questionnaire, with complex routing, so certain questions were only asked based on responses to earlier questions. During 2020 we worked with the YAP online to adapt the survey so it could be administered as a paper administered self-report or online. The YAP made several changes to the survey including the order of sections and wording of questions. Four members of the YAP also received copies of the survey to test how long completion took. The adapted survey was reduced to 84 questions and took between 20 and 30 min to complete when tested by the YAP. The YAP also created and approved patient approach materials and created social media content to promote the study and recruitment. https://www.instagram.com/reel/Co463VgIFUa/?utm_source=ig_web_copy_link&igsh=MzRlODBiNWFlZA== (Accessed on 28 August 2025).

### 2.3. Participants and Setting

BRIGHTLIGHT_2021 opened to recruitment in March 2022; it took until August 2022 for all sites to open. All sites were closed to recruitment in April 2023. Participants were recruited through a direct approach similar to the methods used in the National Cancer Patient Experience Survey (NCPES) and trialed in the 2012 BRIGHTLIGHT cohort [34]. The adaptation to this process was the inclusion of participant review through the TYA MDT to ensure young people were eligible and it was an appropriate time to approach them, i.e., they were not emotionally distressed at that time. A direct approach was used to approach patients as TYA are a disparate group of patients with numerous hematological and solid malignancies. They receive treatment across inpatient, outpatient, and ambulatory care settings in both pediatric and adult services, leading to logistical challenges in recruitment posed by treatment in multiple settings [28,35].

Young people diagnosed between 25 October 2021 and 31 December 2022 were eligible to participate if they were aged 16–24 years at the time of a new cancer diagnosis, were between 4 and 5 months from diagnosis, and being treated in England, Scotland, or Wales. Exclusion criteria included being incapable of completing the survey (e.g., unconscious or mentally incapacitated), having current TYA MDT discussions for recurrence of previous cancer, a high likelihood of death within 6 months as assessed by the TYA MDT, receiving a custodial sentence, and if patients had opted out of having their personal details transferred to Quality Health, the commercial company administering the survey. Additionally, if patients had opted out of having their data used via the National Data Opt-Out scheme, which allows patients to opt out of having their confidential patient information used for research and planning, they did not receive details about the study.

On a monthly basis, young people newly diagnosed with cancer were reviewed for eligibility in the TYA MDT. If there was a reason it was not appropriate to approach a young person at that time, they were reviewed at each subsequent TYA MDT, i.e., screening was dynamic and not a one-off process. Young people initially screened as not eligible continued to be reviewed until they reached 5 months after diagnosis. The TYA MDTs kept detailed screening logs of all patients who were not invited to participate. An anonymized version was reviewed monthly by the research team to ensure recruitment to the study was optimized and to increase our understanding of TYAs’ access to research [25,36,37]. Young people had the opportunity of opting out of receiving study details. To facilitate this and to promote the study, posters explaining the study and the telephone number/email address of a nominated person for young people to contact if they did not want to receive any further information were displayed in prominent areas in the TYA-PTCs and designated hospitals. The posters were also circulated to young people through local means of communication, such as closed hospital Facebook and social media groups.

Following screening for eligibility, a nominated professional in each TYA MDT transferred the names and details of eligible young people to IQVIA, a commercial research company, through a secure server. IQVIA initiated the survey administration process by sending the YAP-developed and approved participant information sheet along with a paper version of the survey and a freepost envelope. Each participant also received a unique QR code and link to complete the survey online if they did not want to complete the paper version. Consistent with established survey methods, two reminders were scheduled for non-responders [38]. The first reminder was sent three weeks after the initial survey pack and included an online survey link. A second reminder was dispatched three weeks after the first reminder, containing another paper copy of the survey, a freepost envelope, and the electronic links to the survey. Return of the survey was implicit of consent. Patients were invited when they were 4–5 months following diagnosis and surveys returned approximately 6 months post diagnosis. The study was approved by an NHS Research Ethics Committee (21/SW/0076) and the Confidentiality Advisory Group for use of patient details without their consent (21/CAG/0108) to enable us to approach participants directly without consent.

### 2.4. Data Collection

The primary outcome for this study was QoL, measured using the PedsQL 4.0™ (Pediatric Quality of Life Questionnaire) [39]. This is validated for young people aged 4–24 years and contains 23 items covering the core domains of physical, emotional, social, and work/school functioning. Responses are on a 5-point Likert scale (never to almost always). A total score summarizes these domains on a 0–100 scale, with 100 representing the best possible QoL. Based on previously published thresholds, a total QoL score of less than 69.7 indicates impairment to quality of life [40].

The survey also included other validated QoL measures similar to the original BRIGHTLIGHT. These included the following:

- EQ-5D (EuroQoL 5 Dimension) is a standardized measure of health status [41]. It comprises 5 dimensions (mobility, self-care, usual activities, pain/discomfort, and anxiety/depression) scored on 3 levels (no, some, severe problems). The EQ-5D visual analog scale records young person’s self-reported health on a scale ranging from ‘best imaginable health state’ to worst imaginable health state’. The higher the score the better the health status.
- Multidimensional Scale of Perceived Social Support (MSPSS) contains 12 statements, which are rated as strongly agree to strongly disagree [42]. Scores are calculated for support by friends, family, and significant others. Total scale scores 1–2.9 are considered low support; a score of 3–5 is considered moderate support; and scores from 5.1 to 7 are considered high support.
- Brief Illness Perception Scale (BIPS) is a measure of the emotional and cognitive representations of illness. It contains eight questions with a fixed response scale specific to each question, e.g., not at all–extremely helpful. Responses are scored 1–10; the higher the score, the greater the perceived illness impact. A total score is calculated through the sum of the scores of the eight questions [43].
- Hospital Anxiety and Depression Scale (HADS) is a measure of the presence and levels of depression and anxiety [44]. It contains 14 items, which are answered on a four-grade verbal scale. Scores of 8–10 are defined as borderline and 11 and over are considered moderate/severe anxiety and depression [45].

### 2.5. Sample Size Calculations and Positivity Assumptions

During study planning we calculated the target sample size. Data from the first wave of data collection from the 2012 BRIGHTLIGHT study provided an estimate of the expected standard deviation of the PedsQL 4.0™ total score of 19.8. To detect a difference in the scores of 8 units (classed as a clinically significant difference [46]) with 90% power required a sample size of at least 732 TYA. This calculation assumed a significance level of 0.01 to allow for multiple comparisons between the three categories of TYA care. The calculation has also allowed for adjustment for confounding factors using a variance inflation factor with a correlation of 0.5. Anticipating we may not meet our planned sample size due to our experience with recruiting TYA to research and to ensure the analysis met the positivity assumption of causal inference methods, a minimum of 10 observations per confounder, per model would predict our minimum sample size needed. With eight confounding variables our minimum sample needed was 80 participants.

### 2.6. Analysis

A mixed effects model was employed to investigate the relationship between the categories of TYA care and different outcomes six months after diagnosis. The model was adjusted for confounding factors identified based on the conceptual model underpinning the BRIGHTLIGHT survey [27] and using a causal diagram in the form of a Directed Acyclic Graph (DAGitty software www.dagitty.net (accessed 28 August 2025); Supplemental Figure S1). Factors adjusted for were gender, age at diagnosis (years), ethnicity (white and other), type of cancer (hematological or solid tumors), prognosis (anticipated 5-year survival <50%, 50–80%, 80–100%), presence of any long-term condition prior to cancer (yes/no), socioeconomic status (Index of Multiple Deprivation), and choice about where to receive treatment (yes/no/doesn’t remember). Geographical location (specified as 11 regions in England) was included in the model as a random effect. These mixed effects models were fitted for the PedsQL 4.0™ total and subdomain scores (physical, social, emotional, school, and psychosocial), HADS, social support score, EQ-5D health status, and illness perception. We obtained estimates for the mean differences in outcome scores between all-TYA-care and joint-care categories compared to the reference category (no-TYA-care). Analyses were carried out following a predefined statistical analysis plan using Stata V.17. To allow a comparison with the 2012 BRIGHTLIGHT study and due to the small numbers of participants from Scotland and Wales, only data from England are reported within the Results Section of this manuscript. Data for the whole cohort along with Scotland and Wales data combined as one cohort can be seen in Supplemental Tables S1–S4.

To examine whether the association between categories of care and patient-reported outcomes varied across sociodemographic groups, we fitted a series of multilevel mixed effects linear regression models. Each outcome (PedsQL domains, EQ-5D index, social support, illness perceptions, anxiety, and depression) was modeled with a random intercept for region. Interaction terms were specified between categories of care and four key covariates: age, gender, ethnicity, and socioeconomic quintile. For each interaction term, we estimated two models: a main effects-only model and a model including the interaction term. Model fit was compared using the Bayesian Information Criterion (BIC). Lower BIC values indicate superior fit, with BIC placing a stronger penalty on model complexity. Following conventional thresholds, negative differences in BIC between the interaction and main effects models were interpreted as evidence in favor of the interaction, while positive differences indicated poorer fit.

## 3. Results

**Figure 1:**
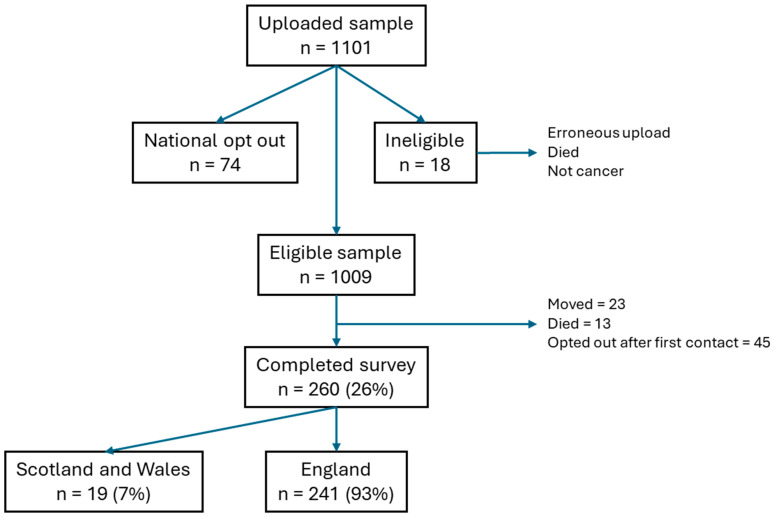
Summary of screening and recruitment.

Across England, Scotland, and Wales a total of 1009 participants were sent the BRIGHTLIGHT survey—260 young people responded (England n = 241; Scotland and Wales n = 19) resulting in a response rate of 25.8% (Figure 1). The survey return mode was recorded for participants in England and Wales (n = 250); 89 surveys were completed online (35.6%) and 161 (64.4%) were returned by post.

Of the 241 participants from England, the category of care could not be assigned to 1 participant due to a missing place of care. Therefore, 240 participants were included in the analysis, of which 66 (27.5%) received all-TYA-care, 89 (37.1%) had joint-care, and 85 (35.4%) received no-TYA-PTC. The details of the participants can be seen in Table 1, including a summary of variables adjusted for in the analysis. The most notable differences between the categories of care (all-TYA-PTC, joint-care, no-TYA-PTC) were observed in gender distribution, age groups, cancer types, and socioeconomic status. Young people who identified as male were more prevalent in the all-TYA-PTC group (41%) compared with the no-TYA-PTC and joint-care groups (31% and 35%, respectively). A higher proportion of those receiving joint-care were aged 16–18 (44%) compared with the other categories, no-TYA-PTC (28%) and all-TYA-PTC (32%). Variation in care received was observed between cancer types; patients with lymphoma most commonly received all-TYA-PTC (53%), while no-TYA-PTC had the highest proportion of patients with carcinomas (25%). Details of the whole cohort and participants from Scotland and Wales can be seen in Supplemental Tables S1 and S2.

Table 2 shows the unadjusted mean summaries of the scores for all outcome measures across different categories of care. Across all categories of care, mean total QoL scores were below 69.7, the recognized threshold for impaired QoL. Around a third (30%) of patients reported moderate/severe anxiety and this was similar across categories of care, while 11% of patients reported moderate/severe depression with some variation across categories of care (HADS score ≥11). Social support was also low across all categories of care (low support are MSPSS scores 1–2.9). Details of the whole cohort and participants from Scotland and Wales can be seen in Supplementary Tables S3 and S4.

Table 3 shows the results of the adjusted mixed effects regression models. The wide confidence intervals spanning zero indicate no statistically significant differences in QoL, social support, anxiety and depression, health status, or illness perception between the categories. In contrast, young people receiving all-TYA-care on average had a lower health status (E5QD index scores) compared with those in the no-TYA-PTC category. This is evidenced by the entire confidence interval lying below zero. Although the magnitude of the effect is small, the narrow interval indicates a reasonably precise estimate, which was not considered to be clinically important. The results based on the whole cohort can be seen in Supplemental Table S5.

Across outcomes, interactions involving gender and socioeconomic quintile consistently improved model fit for health-related quality of life (HRQoL) measures. Specifically, for the total PedsQL score and the physical, emotional, school, and psychosocial functioning domains, the inclusion of a gender × care interaction lowered the BIC, indicating that the effect of care differed by gender. Similarly, the quintile × type of care interaction improved fit for the total PedsQL and several domains (physical, emotional, and school). The social functioning domain showed only a small improvement with gender, and negligible evidence for other interactions.

By contrast, interactions with ethnicity or age did not improve fit for any PedsQL domain and consistently worsened the BIC, suggesting no evidence of the modification of care effects by these variables. For non-PedsQL outcomes (health status, social support, anxiety, and depression), none of the tested interactions improved model fit; in fact, BIC differences were strongly positive, indicating poorer fit when interactions were included. A marginal improvement was observed for the quintile × care interaction in the brief illness perception outcome, although this effect was minimal compared with the robust effects seen for HRQoL (Supplemental Table S6).

## 4. Discussion

We set out to determine if the results generated by the original BRIGHTLIGHT cohort study, which recruited patients between 2012 and 2014, would be similar for patients receiving care across England between October 2021 and December 2022. We theorized that, given the evolution of TYA services and increased coordination of care over the last decade, outcomes for those receiving care in more than one hospital would be improved. Using a direct approach to patient recruitment, BRIGHTLIGHT_2021 aimed to examine differences in patient-reported outcomes between patients receiving care in three different environments. We used patient-reported place of care to categorize those who received all their care in specialist TYA units (all-TYA-PTC), joint care in both TYA-PTCs and other hospitals (joint-care), and those who received no care in TYA-PTCs (no-TYA-PTC). We also recruited patients in Scotland and Wales; however, the numbers recruited were not sufficient to make any meaningful comparisons within or between countries.

After adjustment for confounding factors there were no clinically meaningful differences between the different categories of care in QoL. Similarly, social support, anxiety, depression, and illness perception were similar across the categories of care. In contrast, and similar to the 2012 BRIGHTLIGHT cohort, there were small differences in perceived health status. Young people who accessed a TYA-PTC (all or joint TYA-PTC care) had a lower perceived health status compared with TYA who had no-TYA-PTC care (difference in adjusted means −0.09 and −0.05, respectively). There are several potential reasons which underline this. It may reflect greater education, the promotion of self-care, and the potential impact on their cancer, treatment, and symptoms by healthcare professionals in TYA units; therefore, young people have more awareness of their disease and potential side effects. It may also be underpinned by social comparison theory, where patients compare themselves and their disease status to those around them [47]. In the case of the no-TYA-PTC group, they would have been treated in adult cancer wards/units/outpatient departments surrounded by older or even elderly patients with numerous co-morbidities and illnesses. Young people may perceive themselves to have a relatively better health status in comparison and rate themselves more highly than they really are, a concept known as downward comparison, and has been shown in other cancer studies [48,49,50]. However, the difference between the categories of care was not considered to be clinically meaningful.

Similarly to other reports of QoL in young people following a cancer diagnosis and our 2012 BRIGHTLIGHT study, QoL scores were low regardless of the category of care [27,51]. Alarmingly, QoL scores were lower in BRIGHTLIGHT_2021 compared with the 2012 cohort and this may reflect the recruitment period, which overlapped with the COVID-19 pandemic. Young people were at an increased risk of deterioration of mental health status during the pandemic [52,53,54,55,56,57]. As this was a cross-sectional study, we are unable to speculate what the trajectory of patient-reported outcomes would be within the three categories of care, whether QoL would remain similar between the three groups or whether those in the all-TYA-PTC or joint-care group would experience faster rates of QoL improvement compared with the no-TYA-group, as was the case in the previous BRIGHTLIGHT study [27]. The improvement shown at 6 months after diagnosis suggests this could be sustained, but further long-term studies are required to confirm this.

The 2012 BRIGHTLIGHT study showed that QoL in patients who received some TYA-PTC care was lower than those who had all or none of their care in the TYA-PTC and remained lower throughout the three-year follow-up. We surmised at that time that this may be due to the embryonic nature of some of the TYA services during 2012–2014 and suboptimal coordinated care between PTCs and the networked hospitals. BRIGHTLIGHT_2021 aimed to test this theory that over the past decade coordination between the PTCs and networked hospitals would have improved. This has been reinforced in the service specification with the formalization of a network of care and TYA leadership and workforce working across it. As an observational study, we are unable to conclude on the cause and effect, but increased coordination and communication in care is associated with better outcomes [58], so this is a plausible explanation. However, we also have to acknowledge that our sample size was considerably lower than our target of 732, and the width of the confidence intervals may suggest that with a larger sample size statistically significant differences between categories may have been observed. Therefore, the conclusions and generalizability are limited.

The additional objective of the study was to evaluate a direct approach to recruitment [34]. We found that this did not increase recruitment; the response rate was 25% of the invited eligible invited population and the original method recruited 20% of the eligible diagnosed population [28]. The reason for the low response is unclear; the BRIGHTLIGHT_2021 survey was revised by our patient group, as was the new recruitment method. Based on the current experience, we would not advocate for a direct approach without further investigation of the reasons for low returns. Methods to improve recruitment to research is no. 3 of the top 10 research priorities identified by the James Lind Alliance joint Patient and Professional Partnership, reflecting the need to increase the numbers of TYA in research and generate a robust evidence base to inform care and improve outcomes for TYA [59]. Cancer services have developed over the last decade in England, and this has included increasing access to clinical trials. Most regions now have TYA research champions whose remit is to facilitate access and recruitment to research. Recruiting patients through their clinical teams could now be more successful and it will be interesting to see if this is the case in the future.

Our study had several limitations. Firstly, we had a response rate of 26%, which may have introduced response bias. Our response rate of 26% is typical for TYA aged 16–24 years [60,61,62,63], and strategies to improve recruitment to research for this group are internationally recognized. Our study further highlights the criticality of this if we are to generate an evidence base for care that improves outcomes for this group. To account for smaller numbers, we adopted a causal inference method with an emphasis on the size and direction of the effect estimates. We required 10 young people per confounder per model [64,65,66] and therefore our sample was sufficient. Secondly, the method of recruitment to BRIGHTLIGHT_2021 involved patients being discussed at the TYA clinical meeting at each of the 13 TYA-PTCs in England, and therefore by default, all patients were known to the TYA team and could potentially have had some TYA care delivered outside of the adult unit even though they were classified as having no-TYA-care. Thirdly, the review of the screening logs indicated there were just over ≈ 1400 young people screened during the recruitment period. As there are 2400 new diagnoses in young people every year, this was considerably lower than anticipated. This could partially be explained by not all centers being open during the whole recruitment window, and TYA MDTs may potentially have reviewed fewer young people across their networks than they estimated during the study design. Fourthly, the results of the two BRIGHTLIGHT studies may not be directly comparable due to differences in methodology. We were unable to obtain NHS Hospital Episode Statistics (HES) data due to changes within the NHS Cancer Registry, so patient-reported place of care was used. The analysis of the 2012 BRIGHTLIGHT study using patient-reported place of care generated similar results to those using the HES data. Finally, there is a possibility that the outcome measures were not sensitive enough or did not accurately measure the nuances that specialist TYA care offers to outcome. Future research is needed to understand the mechanism of impact to be able to define specific outcomes that need to be measured to evaluate specialist TYA cancer care. While our study had several limitations, the strengths included the following: a developmentally homogenous age range, national representation, altruistic participation, which reduces response bias (i.e., not incentivized to take part), and increased autonomy with the decision to participate, as invites were sent directly to young people from an independent organization so there was also no healthcare professional gatekeeping. We therefore feel our study adds further to the evidence in support of dedicated cancer care for young people.

## 5. Conclusions

The international attention that TYA have garnered over the past three decades has resulted in numerous models of specialist care [12,13]. Qualitative studies continue to report the value of specialist care for young people with cancer; however, the quantitative evaluation of the benefits has so far been lacking. The BRIGHTLIGHT_2021 study showed no evidence of differences in outcomes related to care delivered between TYA and non-TYA services in England and this is potentially related to ‘enhanced’ joint care. Gender and socioeconomic status influenced how care related to QoL. These findings highlight the need to make services more responsive to social and gender differences among young people with cancer. Alternative methods should be explored for future evaluations to determine the benefits of specialist TYA cancer care, especially the value of a TYA workforce who can bridge between organizations that do not have a specialist TYA environment. These need to be more nuanced to address the complex and geographically variable nature of services and the difficulties in recruiting TYA to studies to ensure an adequate sample size. Exploring the long-term benefits of specialist care is warranted.

## Figures and Tables

**Table 1:** Characteristics of BRIGHTLIGHT _2021 participants (England only) (table reports number (%) unless specified otherwise).

			Categories of Care *		
		Full Sample	All-TYA-PTC	Joint Care	No-TYA-PTC
		n = 241	n = 66 (28%)	n = 89 (37%)	n = 85 (35%)
Gender	Male	85 (35%)	27 (41%)	31 (35%)	26 (31%)
	Female	135 (56%)	34 (52%)	51 (57%)	50 (59%)
	Other	21 (9%)	5 (8%)	7 (8%)	9 (11%)
Age (years)	Mean (SD)	20.00 (2.69)	19.89 (2.59)	19.00 (2.70)	20.07 (2.68)
Age groups	16–18 years	85 (35%)	21 (32%)	39 (44%)	24 (28%)
	19–24 years	156 (65%)	45 (68%)	50 (56%)	61 (72%)
Ethnicity	White	199 (83%)	53 (80%)	75 (84%)	70 (82%)
	Other **	42 (17%)	13 (20%)	14 (16%)	15 (18%)
Socioeconomic status (IMD quintile)	1—most deprived	65 (27%)	29 (44%)	17 (19%)	19 (22%)
	2	30 (12%)	5 (8%)	13 (15%)	12 (14%)
	3	75 (31%)	17 (26%)	35 (39%)	23 (27%)
	4	22 (9%)	5 (8%)	9 (10%)	7 (8%)
	5—least deprived	32 (13%)	8 (12%)	8 (9%)	16 (19%)
	Missing	17 (7%)	2 (3%)	7 (8%)	8 (9%)
Marital status	Single	215 (89%)	58 (88%)	83 (93%)	73 (86%)
	Married/civil partnership	2 (1%)	0 (0%)	2 (2%)	0 (0%)
	Cohabit	21 (9%)	8 (12%)	3 (3%)	10 (12%)
	Divorced	1 (0%)	0 (0%)	0 (0%)	1 (1%)
	Missing	2 (1%)	0 (0%)	1 (1%)	1 (1%)
Current status	In education	25 (11%)	3 (5%)	8 (9%)	14 (17%)
	Working part/ full time	41 (18%)	11 (17%)	13 (15%)	17 (21%)
	Apprenticeship/ unpaid/voluntary	3 (1%)	1 (2%)	1 (1%)	1 (1%)
	Unemployed	19 (8%)	10 (16%)	5 (6%)	4 (5%)
	Long-term sick	15 (6%)	2 (3%)	8 (9%)	5 (6%)
	Not seeking work	60 (26%)	16 (25%)	23 (27%)	20 (24%)
	Missing	2 (1%)	1 (2%)	1 (1%)	0 (0%)
Region	London	55 (23%)	15 (23%)	21 (24%)	18 (21%)
	East Midlands	18 (7%)	8 (12%)	2 (2%)	8 (9%)
	East of England	29 (12%)	10 (15%)	9 (10%)	10 (12%)
	Merseyside	17 (7%)	6 (9%)	9 (10%)	2 (2%)
	Northeast	15 (6%)	2 (3%)	5 (6%)	8 (9%)
	Northwest	20 (8%)	5 (8%)	7 (8%)	8 (9%)
	Southwest	24 (10%)	1 (2%)	15 (17%)	8 (9%)
	Thames Valley	16 (7%)	0 (0%)	8 (9%)	8 (9%)
	Wessex	8 (3%)	1 (2%)	4 (4%)	3 (4%)
	West Midlands	10 (4%)	4 (6%)	2 (2%)	4 (5%)
	Yorkshire	29 (12%)	14 (21%)	7 (8%)	8 (9%)
Cancer type	Leukemia	20 (8%)	8 (12%)	7 (8%)	4 (5%)
	Lymphoma	89 (37%)	35 (53%)	26 (29%)	28 (33%)
	CNS	15 (6%)	5 (8%)	5 (6%)	5 (6%)
	Bone and Soft Tissue Sarcoma	20 (8%)	2 (3)	9 (10%)	9 (11%)
	Germ Cell	35 (15%)	10 (15)	18 (20%)	7 (8%)
	Melanoma	11 (5%)	1 (2)	4 (4%)	6 (7%)
	Carcinomas (not skin)	42 (17%)	5 (8)	16 (18%)	21 (25%)
	Other	9 (4%)	0 (0)	4 (4%)	5 (6%)
Prognosis	80–100%	186 (77%)	53 (80%)	67 (75%)	66 (78%)
	50–80%	45 (19%)	12 (18%)	17 (19%)	15 (18%)
	<50%	4 (2%)	1 (2%)	2 (2%)	1 (1%)
	Missing	6 (2%)	0 (0%)	3 (3%)	3 (4%)
Long-term health condition	No	198 (82%)	57 (86%)	71 (80%)	69 (81%)
	Yes	42 (17%)	9 (14%)	17 (19%)	16 (19%)
	Missing	1 (0%)	0 (0%)	1 (1%)	0 (0%)
Choice of care	Yes	106 (44%)	29 (44%)	38 (43%)	39 (46%)
	No	106 (44%)	28 (42%)	44 (49%)	34 (40%)
	Can’t remember	27 (11%)	9 (14%)	6 (7%)	12 (14%)
	Missing	2 (1%)	0 (0%)	1 (1%)	0 (0%)

* Of 241 participants from England, category of care could not be assigned to 1 participant due to missing place of care; ** Other includes Asian or Asian British; Black or Black British; Mixed/multiple ethnic groups; or other ethnic group. Categories were collapsed due to small numbers. CNS: central nervous system; SD: standard deviation; IMD: Index of Multiple Deprivation. Percentages may not add up to 100% due to rounding.

**Table 2:** Summary of outcome scores by categories of care 6 months following diagnosis (England only).

	Total			Categories of Care *								
				All-TYA-PTC			Joint Care			No-TYA-PTC		
	241 (100%)			66 (28%)			89 (37%)			85 (35%)		
	n	Mean	SD	n	Mean	SD	n	Mean	SD	n	Mean	SD
**Total QoL score**	*238*	58.65	20.13	*65*	56.47	21.71	*89*	57.92	19.13	*84*	61.10	19.89
Physical function	*237*	55.58	27.21	*65*	50.85	29.11	*88*	55.58	26.50	*84*	59.24	26.15
Emotional function	*240*	54.82	23.03	*66*	53.26	24.57	*89*	54.33	22.16	*85*	56.56	22.87
Social function	*239*	73.43	22.24	*65*	73.79	22.37	*89*	71.69	22.18	*85*	74.98	22.36
Work/school/college/ university function	*219*	51.00	26.51	*59*	47.99	28.59	*80*	50.45	26.23	*80*	53.77	25.23
Psychosocial summary score	*240*	59.77	19.88	*66*	58.68	21.12	*89*	58.67	18.54	*85*	61.79	20.33
**Health status**	*238*	0.67	0.27	*66*	0.61	0.31	*89*	0.67	0.26	*83*	0.73	0.23
**Social support**	*231*	1.83	0.81	*65*	1.77	0.77	*85*	1.89	0.89	*81*	1.81	0.74
**Illness perception**	*200*	36.55	11.87	*51*	35.57	11.85	*76*	36.76	10.84	*73*	37.00	12.99
Anxiety **	230	8.40	4.43	62	8.35	4.43	87	8.44	4.48	81	8.41	4.42
Borderline n (%)	54 (23.48)			11 (17.67)			20 (22.99)			23 (28.40)		
Severe n (%)	70 (30.43)			19 (30.65)			26 (29.89)			25 (30.86)		
Depression **	237	5.88	3.84	65	6.95	4.08	89	5.25	3.55	83	5.71	3.83
Borderline n (%)	48 (20.25)			18 (27.69)			17 (19.10)			13 (15.66)		
Severe n (%)	26 (10.97)			9 (13.85)			7 (7.87)			10 (12.05)		

* Of 241 participants from England, category of care could not be assigned to 1 participant due to missing place of care. ** Severe anxiety and depression defined as HADS-A or HADS-D score ≥11, respectively. QoL: quality of life.

**Table 3:** Results from confounder-adjusted mixed models multivariable regression investigating the relationship between categories of TYA care received and outcomes 6 months after diagnosis (England only, n = complete responses analyzed).

		Adjusted Difference in Means	95% Confidence Interval	*p*-Value †
**Quality of life total score (N = 214)**				
TYA care category (v no-TYA-PTC)	all-TYA-PTC	−2.28	−8.85 to 4.29	0.568
	Joint care	−4.35	−10.34 to 1.63	
**Physical functioning (N = 213)**				
TYA care category (v no-TYA-PTC)	all-TYA-PTC	−4.15	−12.96 to 4.65	0.558
	Joint care	−3.77	−11.80 to 4.24	
**Emotional functioning (N = 216)**				
TYA care category (v no-TYA-PTC)	all-TYA-PTC	−3.96	−11.33 to 3.4	0.299
	Joint care	−5.14	−11.90 to 1.61	
**Social functioning (N = 215)**				
TYA care category (v no-TYA-PTC)	all-TYA-PTC	1.59	−6.07 to 9.25	0.291
	Joint care	−4.17	−11.14 to 2.78	
**Work/school/college/university functioning (N = 197)**				
TYA care category (v no-TYA-PTC)	all-TYA-PTC	−2.49	−11.45 to 6.45	0.584
	Joint care	−4.39	−12.71 to 3.93	
**Psychosocial summary score (N = 216)**				
TYA care category (v no-TYA-PTC)	all-TYA-PTC	−1.47	−7.98 to 5.02	0.521
	Joint care	−4.52	−10.49 to 1.44	
**Health status (N = 214)**				
TYA care category (v no-TYA-PTC)	all-TYA-PTC	−0.09	−0.18 to −0.01	0.099
	Joint care	−0.05	−0.14 to 0.02	
**Social support (N = 207)**				
TYA care category (v no-TYA-PTC)	all-TYA-PTC	−0.12	−0.39 to 0.15	0.125
	Joint care	0.09	−0.16 to 0.35	
**Illness perception (N = 179)**				
TYA care category (v no-TYA-PTC)	all-TYA-PTC	−1.72	−6.31 to 2.86	0.577
	Joint care	0.73	−3.22 to 4.69	
**Anxiety (N = 208)**				
TYA care category (v no-TYA-PTC	all-TYA-PTC	−0.03	−1.43 to 1.51	0.852
	Joint care	−0.36	−0.98 to 1.7	
**Depression (N = 213)**				
TYA care category (v no-TYA-PTC)	all-TYA-PTC	−0.74	−0.55 to 2.04	0.185
	Joint care	0.47	−1.65 to 0.7	

† Likelihood ratio test.

## Data Availability

Further details of the BRIGHTLIGHT program of work are available through the study website (www.brightlightstudy.com). Data that is not held under license with NHS England will be available from late 2025 when the primary analysis is complete. We welcome collaboration; for general data sharing enquiries please contact RMT (rtaylor13@nhs.net).

## Supplementary Materials

The following supporting information can be downloaded at: https://www.mdpi.com/article/10.3390/cancers17233868/s1, Figure S1: Matrix representing the causal diagram explaining confounding variable to enter in the Directed Acyclic Graph (DAG); Table S1: Characteristics of BRIGHTLIGHT_2021 participants, England, Scotland and Wales (n; %); Table S2: Characteristics of BRIGHTLIGHT_2021, Scotland and Wales only (n); Table S3: Comparison of summary of outcome scores mean outcomes, standard deviations and sample between by three Categories of Care 6-months following diagnosis, England, Scotland and Wales; Table S4: Comparison of Summary of outcome scores, mean outcomes and standard deviations by three Categories of Care 6-months following diagnosis, Scotland and Wales only; Table S5: Results from mixed models investigating the relationship between categories of TYA care received and outcomes 6-months after diagnosis for the full cohort; Table S6: Comparison of goodness-of-fit for models including various interaction terms.
