# Supplementary material for: Does Joint Care Impact Teenage and Young Adult’s Patient-Reported Outcomes After a Cancer Diagnosis? Results from BRIGHTLIGHT_2021
Source: Cancers (Basel). 2025 Dec 2;17(23):3868. doi: 10.3390/cancers17233868 (PMC12691178; doi:10.3390/cancers17233868)
Supplement: Supplementary file 1 [file cancers-17-03868-s001.zip › cancers-3871605-supplementary.pdf]

Supplementary File

Figure S1: Matrix representing the causal diagram explaining confounding variable to enter in the Directed Acyclic Graph (DAG)

	TYA SCL	QOL	Age	Choice	Prg	CT	Ethnicity	Gender	Geography	I&C	LTC	Rt D	SE	SES	SS
TYA SCL		⇗	⇙	⇙	⇙	⇙	⇙	O	⇙	⇗	⇙	⇙	⇗	⇙	⇗
QOL			⇙	⇙	⇙	⇙	⇙	⇙	⇙	⇙	⇙	⇙	⇙	⇙	⇙
Age				⇗	O	⇗	O	O	O	⇗	⇗	⇗	⇗	⇗	⇗
Choice					⇙	⇙	⇙	O	⇙	⇗	⇙	⇙	⇗	⇙	⇗
Prg						⇙	⇙	O	⇙	⇗	⇙	⇙	⇗	⇙	⇗
CT							⇙	⇙	⇙	⇗	⇙	⇗	⇗	⇙	⇗
DoH							⇙	⇙	⇙	⇗	⇙	⇙	⇗	⇙	⇗
Ethnicity								O	⇙	⇗	⇗	⇗	⇗	⇗	⇗
Gender									O	⇗	⇗	⇗	⇗	O	⇗
Geography										⇗	⇗	⇗	⇗	⇗	⇗
I&C											⇙	⇙	⇗	⇙	⇗
LTC												⇗	⇗	⇙	⇗
RtD													⇗	⇙	⇗
SE														⇙	⇗
SES															⇗
SS															

CT: cancer type; I&C: information & communication; LTC: long-term condition; Prg: prognosis; QOL: quality of life; RtD: route to diagnosis; SE: symptom experience; SES: socioeconomic status; SS: social support
O – indicates a null relationship

Table S1: Characteristics of BRIGHTLIGHT_2021 participants, England, Scotland and Wales (n; %)

		Categories of care			
	**Total**	**all-TYA-PTC**	**Joint care**	**no-TYA-PTC**	**Missing**
	n=260	n=75 (29%)	n=93 (36%)	n=90 (35%)	n=2 (1%)
**Gender**					
Male	94 (36)	34 (45)	32 (34)	27 (30)	1 (50)
Female	145 (56)	36 (48)	54 (58)	54(60)	1 (50)
Other	21 (8)	5 (7)	7(8)	9 (10)	0 (0)
**Age groups**					
16-18 years	94 (36)	24(32)	41(44)	27 (30)	2(100)
19-25 years	166 (64)	51(68)	52 (56)	63(70)	0 (0)
**Marital status**					
Married/civil partnership	2 (1)	0 (0)	2 (2)	0 (0)	0 (0)
Cohabit	24 (9)	10 (13)	3 (3)	11 (12)	0 (0)
Divorced	1 (1)	0 (0)	0 (0)	1 (1)	0 (0)
Single	231 (89)	65 (87)	87 (94)	77 (86)	2 (100)
Missing	2 (1)	0 (0)	1 (1)	1 (1)	0 (0)
**Region**					
London	55 (21)	15 (20)	21 (23)	18 (20)	1 (50)
East Midlands	18 (7)	8 (11)	2 (2)	8 (9)	0 (0)
East of England	29 (11)	10 (13)	9 (10)	10 (11)	0 (0)
Merseyside	17 (7)	6 (8)	9 (10)	2 (2)	0 (0)
Northeast	15 (6)	2 (3)	5 (5)	8 (9)	0 (0)
Northwest	20 (8)	5 (7)	7 (8)	8 (9)	0 (0)
Southwest	24 (9)	1 (1)	15 (16)	8 (9)	0 (0)
Thames Valley	16 (6)	0 (0)	8 (9)	8 (9)	0 (0)
Wales	9 (3)	4 (5)	2 (2)	2 (2)	1 (50)
Wessex	8 (3)	1 (1)	4 (4)	3 (3)	0 (0)
West Midlands	10 (4)	4 (5)	2 (2)	4 (4)	0 (0)

Yorkshire	29 (1)	14 (19)	7 (8)	8 (9)	0 (0)
Scotland	10 (4)	5 (7)	2 (2)	3 (3)	0 (0)
**Cancer type**					
Leukaemia	22 (8)	10 (13)	7 (8)	4 (4)	1 (50)
Lymphoma	96 (37)	39 (52)	27 (29)	30 (33)	0 (0)
CNS	15 (6)	5 (7)	5 (5)	5 (6)	0 (0)
Bone tumours	13 (5)	1 (1)	7 (8)	5 (6)	0 (0)
Soft Tissue Sarcoma	10 (4)	2 (3)	4 (4)	4 (4)	0 (0)
Germ Cell	37 (14)	12 (16)	18 (19)	7 (8)	0 (0)
Melanoma	11 (4)	1 (1)	4 (4)	6 (7)	0 (0)
Carcinomas (not skin)	46 (18)	5 (7)	17 (18)	23 (26)	1 (50)
Other	10 (4)	0 (0)	4 (4)	6 (7)	0 (0)
**Prognosis**					
50-80	52 (20)	15 (20)	19 (20)	17 (19)	1 (50)
80-100	198 (76)	59 (79)	69 (74)	69 (77)	1 (50)
<50	4 (2)	1 (1)	2 (2)	1 (1)	0 (0)
Missing	6 (2)	0 (0)	3 (3)	3 (3)	0 (0)
**Long term health condition**					
Yes	45 (17)	10 (13)	17 (18)	18 (20)	0 (0)
No	214 (82)	65 (87)	75 (81)	72 (80)	2 (100)
Missing	1 (>1)	0 (0)	1 (1)	0 (0)	0 (0)
**Ethnicity**					
White	215 (83)	61 (81)	79 (85)	73 (81)	2(100)
Other*	45 (17)	14 (16)	14 (15)	17 (19)	0 (0)
**Socioeconomic status (IMD quantile)**					
1 - most deprived	65 (25)	29 (39)	17 (18)	19 (21)	0 (0)
2	30 (12)	5 (7)	13 (14)	12 (13)	0 (0)
3	75 (29)	17 (23)	35 (38)	23 (26)	0 (0)
4	22 (8)	5 (7)	9 (10)	7 (8)	1 (50)

5 - least deprived	32 (12)	8 (11)	8 (9)	16 (18)	0 (0)
Missing	36 (14)	11 (15)	11 (12)	13 (14)	1 (50)
**Current status**					
In education	27 (11)	5 (7)	8 (9)	14 (16)	0 (0)
Working part/full time	42 (17)	12 (16)	13 (15)	17 (20)	0 (0)
Apprenticeship/unpaid/voluntary	3 (1)	1 (1)	1 (1)	1 (1)	0 (0)
Unemployed	22 (9)	11 (15)	6 (7)	5 (6)	0 (0)
Long-term sick	16 (6)	2 (3)	9 (10)	5 (6)	0 (0)
Not seeking work	66 (27)	20 (27)	24 (27)	21 (24)	1 (100)
Missing	4 (2)	2 (3)	1 (1)	1 (1)	0 (0)
**Choice of care**					
Yes	111 (43)	31 (41)	40 (43)	40 (44)	0 (0)
No	117 (45)	34 (45)	46 (49)	37 (41)	0 (0)
Can’t remember	29 (11)	10 (13)	6 (6)	13 (14)	0 (0)
Missing	3 (1)	0 (0)	1 (1)	0 (0)	2 (100)

Table S2: Characteristics of BRIGHTLIGHT_2021, Scotland and Wales only (n)

	Total n = 19		All-TYA-PTC n = 9 (47)		Joint care n = 4 (21)		no-TYA-PTC n = 5 (26)		Missing n = 1 (5)	
**Gender**										
Male	9	(47)	7	(78)	1	(25)	1	(20)	0	(0)
Female	10	(53)	2	(22)	3	(75)	4	(80)	1	(100)
**Age groups**										
13-18	9	(47)	3	(33)	2	(50)	3	(60)	1	(100)
19-24 years	10	(53)	6	(67)	2	(50)	2	(40)	0	(0)
**Marital status**										
Cohabit	3	(16)	2	(22)	0	(0)	1	(20)	0	(0)
Single	16	(84)	7	(78)	4	(100)	4	(80)	1	(100)
**Region**										
Wales	9	(47)	4	(44)	2	(50)	2	(40)	1	(100)
Scotland	10	(53)	5	(56)	2	(50)	3	(60)	0	(0)
**Cancer type**										
Leukaemia	2	(11)	2	(22)	0	(0)	0	(0)	0	(0)
Lymphoma	7	(37)	4	(44)	1	(25)	2	(40)	0	0)
Bone Tumours	3	(16)	1	(11)	2	(50)	0	(0)	0	0)
Germ Cell	2	(11)	2	(22)	0	(0)	0	(0)	0	0)
Carcinomas (not skin)	4	(21)	0	(0)	1	(25)	2	(40)	1	100)
Other	1	(5)	0	(0)	0	(0)	1	(20)	0	0)
**Prognosis**										
50-80%	7	(37)	3	(33)	2	(50)	2	(40)	0	0)
80-100%	12	(63)	6	(67)	2	(50)	3	(60)	1	100)
**Longterm Condition**										
Yes	3	(16)	1	(11)	0	(0)	2	(40)	0	0)
No	16	(84)	8	(89)	4	(100)	3	(60)	1	100)

**Ethnicity**										
White	16	(84)	8	(89)	4	(100)	3	(60)	1	100)
Other	3	(16)	1	(11)	0	(0)	2	(40)	0	0)
**Employment**										
In education	8	(42)	2	(22)	3	(75)	2	(40)	1	(100)
Working part/full time	2	(11)	2	(22)	0	(0)	0	(0)	0	(0)
Apprenticeship/unpaid/voluntary	1	(5)	0	(0)	0	(0)	1	(20)	0	(0)
Unemployed	3	(16)	1	(11)	1	(25)	1	(20)	0	(0)
Long-term sick	5	(26)	4	(44)	0	(0)	1	(20)	0	(0)
Not seeking work	0	(0)	0	(0)	0	(0)	0	(0)	0	(0)
Missing	0	(0)	0	(0)	0	(0)	0	(0)	0	(0)
**Choice of care**										
Yes	5	(26)	2	(22)	2	(50)	1	(20)	0	(0)
No	11	(58)	6	(67)	2	(50)	3	(60)	0	(0)
Can't remember	2	(11)	1	(11)	0	(0)	1	(20)	0	(0)
Missing	1	(5)	0	(0)	0	(0)	0	(0)	100	(1)

Table S3: Comparison of summary of outcome scores mean outcomes, standard deviations and sample between by three Categories of Care 6-months following diagnosis, England, Scotland and Wales

	Total n = 260			all-TYA-PTC n = 75 (29%)			Joint care n = 93 (36%)			no-TYA-PTC n = 90 (35%)		
	n	**Mean**	**SD**	*n*	**Mean**	**SD**	*n*	**Mean**	**SD**	*n*	**Mean**	**SD**
**Total QoL score**	*256*	58.38	20.42	*74*	58.07	21.89	*93*	57.15	19.40	*89*	59.91	20.33
Physical function	*255*	55.11	27.41	*74*	51.93	29.10	*92*	54.79	27.12	*89*	58.09	26.24
Emotional function	*258*	54.78	23.63	*75*	55.80	25.73	*93*	53.71	22.10	*90*	55.03	23.55
Social function	*257*	73.46	22.22	*74*	75.22	21.98	*93*	71.03	22.18	*90*	74.53	22.47
Work/school/college function	*236*	50.27	27.08	*67*	49.20	29.28	*84*	49.30	26.50	*85*	52.07	26.07
Psychosocial summary score	*258*	59.57	20.20	*75*	60.40	21.34	*93*	57.90	18.70	*90*	60.59	20.83
**Health status**	*246*	0.67	0.27	*70*	0.61	0.31	*91*	0.67	0.26	*85*	0.72	0.23
**Social support**	*248*	1.80	0.80	*74*	1.70	0.76	*88*	1.89	0.88	*86*	1.81	0.73
**Illness perception**	*217*	36.73	11.84	*59*	35.22	11.68	*80*	37.20	11.02	*78*	37.40	12.79
**Anxiety**	*248*	8.52	4.47	*71*	8.15	4.35	*91*	8.66	4.55	*86*	8.67	4.52
Borderline n (%)		57 (21)			13 (17)			21 (23)			23 (26)	
Severe n (%)		78 (30)			20 (27)			29 (31)			29 (32)	
**Depression**	*255*	5.92	3.88	*74*	6.58	4.06	*93*	5.41	3.61	*88*	5.91	3.98
Borderline n (%)		50 (19)			19 (25)			17 (18)			14 (16)	
Severe n (%)		30 (12)			9 (12)			9 (10)			12 (13)	

Table S4: Comparison of Summary of outcome scores, mean outcomes and standard deviations by three Categories of Care 6-months following diagnosis, Scotland and Wales only

	Total n = 19			all-TYA-PTC n = 9 (50%)			Joint care n = 4 (22%)			no-TYA-PTC n = 5 (28%)		
	n	**Mean**	**SD**	*n*	**Mean**	**SD**	*n*	**Mean**	**SD**	*n*	**Mean**	**SD**
**Total QoL score**	*18*	54.78	24.25	*9*	69.59	20.72	*4*	40.00	20.02	*5*	39.94	18.79
Physical function	*18*	48.96	30.16	*9*	59.72	29.50	*4*	37.50	39.11	*5*	38.75	21.38
Emotional function	*18*	54.17	31.35	*9*	74.44	27.89	*4*	40.00	17.80	*5*	29.00	21.62
Social function	*18*	73.89	22.46	*9*	85.56	16.48	*4*	56.25	18.87	*5*	67.00	25.64
Work/school/college function	*17*	40.88	33.13	*8*	58.13	34.74	*4*	26.25	23.94	*5*	25.00	26.93
Psychosocial summary score	*18*	56.81	24.55	*9*	73.06	19.54	*4*	40.83	15.43	*5*	40.33	20.73
**Health status**	*8*	0.54	0.32	*4*	0.59	0.31	*2*	0.52	0.47	*2*	0.46	0.43
**Social support**	*17*	1.47	0.53	*9*	1.19	0.47	*3*	1.92	0.17	*5*	1.70	0.54
**Illness perception**	*17*	38.94	11.55	*8*	33.00	10.95	*4*	45.50	12.71	*5*	43.20	8.14
**Anxiety**	*18*	10.00	4.91	*9*	6.78	3.70	*4*	13.50	3.70	*5*	13.00	4.24
Borderline n (%)		*2 (11)*			*2 (22)*			*1 (25)*			*0 (0)*	
Severe n (%)		*1 (5)*			*1 (11)*			*3 (75)*			*4 (80)*	
**Depression**	*18*	6.50	4.45	*9*	3.89	2.76	*4*	9.00	3.46	*5*	9.20	5.40
Borderline n (%)		*1 (5)*			*1 (11)*			*0 (0)*			*1 (20)*	
Severe n (%)		*0 (0)*			*0 (0)*			*2 (50)*			*2 (40)*	

Table S5: Results from mixed models investigating the relationship between categories of TYA care received and outcomes 6-months after diagnosis for the full cohort *

Outcomes	Adjusted difference in means		95% Confidence Interval	P-value †
**Quality of life total score (n= 248)**				
TYA care category	all-TYA-PTC	-2.26	-8.37 to 3.85	0.59
(vs no-TYA-PTC)	Joint care	-2.86	-8.56 to 2.83	
**Physical functioning (n= 247)**				
TYA care category	all-TYA-PTC	-6.09	-14.29 to 2.10	0.34
(vs no-TYA-PTC)	Joint care	-3.14	-10.78 to 4.50	
**Emotional functioning (n= 250)**				
TYA care category	all-TYA-PTC	-1.43	-8.44 to 5.59	0.94
(vs no-TYA-PTC)	Joint care	-2.10	-8.67 to 4.47	
**Social functioning (n= 249)**				
TYA care category	all-TYA-PTC	0.79	-6.09 to 7.66	0.36
(vs no-TYA-PTC)	Joint care	-3.77	-10.18 to 2.64	
**Work/school/college functioning (n= 228)**				
TYA care category	all-TYA-PTC	-2.60	-10.99 to 5.80	0.79
(vs no-TYA-care)	Joint care	-2.30	-10.19 to 5.59	
**Psychosocial summary score (n= 250)**				
TYA care category	all-TYA-PTC	-0.80	-6.83 to 5.23	0.61
(vs no-TYA-care)	Joint care	-2.82	-8.47 to 2.83	
**Health status (n= 214)**				
TYA care category	all-TYA-PTC	-0.10	-0.18 to -0.01	0.05
(vs no-TYA-care)	Joint care	-0.06	-0.14 to 0.02	
**Social support (n= 240)**				
TYA care category	all-TYA-PTC	-0.03	-0.28 to 0.21	0.31
(vs no-TYA-care)	Joint care	0.09	-0.14 to 0.32	
**Illness perception (n= 210)**				
TYA care category	all-TYA-PTC	-1.24	-5.33 to 2.84	0.77
(vs no-TYA-care)	Joint care	0.20	-3.48 to 3.88	
**Anxiety (n=240)**				
TYA care category	all-TYA-PTC	0.13	-1.24 to 1.49	0.97
(vs no-TYA-care)	Joint care	1403816.00	-1.12 to 1.40	
**Depression (n=247)**				
TYA care category	all-TYA-PTC	0.88	-0.32 to 2.08	0.09
(vs no-TYA-care)	Joint care	-0.48	-1.60 to 0 .64	

*Model does not contain IMD: Index of Multiple Deprivation as we do not have this for Scotland and Wales.

Table S6: Comparison of goodness-of-fit for models including various interaction terms

	Interaction		Main effects model	Main effects and interaction term model	Difference
**Total PEDSQL**	TYA categories of care and	Age	2096.236	2097.575	1.34
	TYA categories of care and	Gender	2096.236	2087.489	-8.75
	TYA categories of care and	Ethnicity	2096.236	2114.721	18.49
	TYA categories of care and	Quintile	2096.236	2091.021	-5.21
PEDSQL (Physical)	TYA categories of care and	Age	2218.717	2223.83	5.11
	TYA categories of care and	Gender	2218.717	2199.785	-18.93
	TYA categories of care and	Ethnicity	2218.717	2245.773	27.06
	TYA categories of care and	Quintile	2218.717	2213.191	-5.53
PEDSQL (Emotional)	TYA categories of care and	Age	2172.314	2177.544	5.23
	TYA categories of care and	Gender	2172.314	2161.07	-11.24
	TYA categories of care and	Ethnicity	2172.314	2188.441	16.13
	TYA categories of care and	Quintile	2172.314	2165.915	-6.40
PEDSQL (Social)	TYA categories of care and	Age	2161.247	2172.333	11.09
	TYA categories of care and	Gender	2161.247	2158.581	-2.67
	TYA categories of care and	Ethnicity	2161.247	2190.275	29.03
	TYA categories of care and	Quintile	2161.247	2162.699	1.45
PEDSQL (School)	TYA categories of care and	Age	2035.271	2038.96	3.69
	TYA categories of care and	Gender	2035.271	2027.796	-7.47
	TYA categories of care and	Ethnicity	2035.271	2057.18	21.91
	TYA categories of care and	Quintile	2035.271	2029.579	-5.69
PEDSQL (Psychosocial)	TYA categories of care and	Age	2107.57	2114.327	6.76
	TYA categories of care and	Gender	2107.57	2103.658	-3.91
	TYA categories of care and	Ethnicity	2107.57	2134.294	26.72
	TYA categories of care and	Quintile	2107.57	2108.05	0.48
**Health status**	TYA categories of care and	Age	222.032	244.3863	22.35
	TYA categories of care and	Gender	222.032	239.7507	17.72
	TYA categories of care and	Ethnicity	222.032	236.1678	14.14
	TYA categories of care and	Quintile	222.032	233.3169	11.28
**Social support**	TYA categories of care and	Age	286.3342	306.9405	20.61
	TYA categories of care and	Gender	286.3342	321.2683	34.93
	TYA categories of care and	Ethnicity	286.3342	303.8037	17.47
	TYA categories of care and	Quintile	286.3342	302.6765	16.34
**Brief illness**	TYA categories of care and	Age	1513.498	1592.404	78.91
	TYA categories of care and	Gender	1513.498	1579.228	65.73
	TYA categories of care and	Ethnicity	1513.498	1604.283	90.78
	TYA categories of care and	Quintile	1586.061	1584.429	-1.63
**Anxiety**	TYA categories of care and	Age	1385.674	1395.7	10.03
	TYA categories of care and	Gender	1385.674	1391.157	5.48
	TYA categories of care and	Ethnicity	1385.674	1399.095	13.42
	TYA categories of care and	Quintile	1385.674	1387.4	1.73
**Depression**	TYA categories of care and	Age	1380.04	1391.093	11.05
	TYA categories of care and	Gender	1380.04	1383.136	3.10
	TYA categories of care and	Ethnicity	1380.04	1402.772	22.73

TYA categories of care and	Quintile	1380.04	1382.118	2.08
